# How Important Are Dietary Habits Compared to Other Factors for Sleep Quality?—An Analysis Using Data from a Specific Region in Japan

**DOI:** 10.3390/nu17172787

**Published:** 2025-08-27

**Authors:** Makoto Hazama, Hiroyo Kagami-Katsuyama, Naohito Ito, Mari Maeda-Yamamoto, Jun Nishihira

**Affiliations:** 1 Department of Medical Management and Informatics, Hokkaido Information University, Ebetsu 069-8585, Japan; m-hazama@do-johodai.ac.jp (M.H.);; 2 Institute of Food Research, National Agriculture and Food Research Organization, Tsukuba 305-8642, Japan

**Keywords:** improvement of sleep quality, quantitative comparison of contributing factors, dietary habits, dynamic multivariate panel model, structural causal model

## Abstract

**Background/Objectives**: The improvement of sleep quality is unquestionably a critical issue in public health. While numerous factors influence sleep quality, the relative importance of dietary habits remains insufficiently understood. The objective of this study is to evaluate the contribution of dietary habits by quantitatively comparing the effects of various determinants of sleep quality. **Methods**: Using sleep diary data from healthy males and females residing in a specific region of Japan, we estimated a dynamic multivariate panel model (DMPM) to obtain posterior predictive distributions on a scale that allows for comparisons across factor categories. Three outcome variables were adopted to measure sleep quality: presence or absence of daytime drowsiness, ease of falling asleep, and ease of waking up. The determinants of sleep quality examined in the analysis were categorized into six groups: stress factors, bedtime conditions, weather conditions, physical characteristics, exercise habits, and dietary habits. **Results**: The analysis revealed that although there were some seasonal and gender differences, dietary habits showed effect sizes that were no smaller than those of other determinants across all outcome variables. **Conclusions**: These results suggest that improving dietary habits, along with enhancing exercise habits and bedtime conditions, is a valid and equally important strategy for promoting better sleep.

## 1. Introduction

High-quality, adequate sleep plays a vital role in not only fostering and preserving human health but also sustaining daily functioning, promoting longevity, and enriching overall well-being [1,2,3,4,5,6,7,8,9]. It is well known that sleep quality is influenced by a variety of factors, among which dietary habits play an important role. Numerous studies have already examined the relationship between dietary habits and sleep quality [10,11,12,13,14,15,16,17,18,19,20,21]. For instance, intake of foods rich in tryptophan or carbohydrates has been found to improve sleep quality [12,16,20], while consumption of processed foods or free-sugar-rich foods has been negatively correlated with sleep quality [11,17,21]. In addition, there is research on the use of nutraceuticals (e.g., melatonin, magnesium, omega-3 fatty acids, etc.) to enhance sleep quality [22]. Although dietary habits are one of the many factors that affect sleep quality, to the best of our knowledge, no studies have investigated how large their effect is in comparison to other factors. The purpose of this paper is to quantitatively evaluate the relative importance of dietary factors on sleep quality among a wide range of influencing variables.

The primary reason why no study has been found that quantitatively evaluates the relative importance among categories of factors affecting sleep quality is, quite simply, the limitation of available data. Since sleep quality is influenced by a wide range of factors, the data used for analysis must include a rich set of survey items. Furthermore, observational data have inherent limitations when it comes to estimating causal relationships. This paper seeks to address these constraints by utilizing comprehensive survey data and relying on methods of observational causal inference [23].

More concretely, in order to quantitatively compare the determinants of sleep quality by category, this analysis uses sleep diary data covering up to six consecutive days. In the sleep diaries, three items related to sleep quality are treated as outcome variables, while variables identified from prior studies [24,25,26,27,28,29,30,31,32,33,34,35,36,37,38,39,40,41,42,43,44,45,46,47,48,49,50,51,52] as determinants of sleep quality were consolidated into a dataset for analysis. That is, stress-related measures taken before going to bed and upon waking, as well as bedtime conditions such as caffeine intake, serve as explanatory variables. These are then integrated with meteorological data, physical characteristics, exercise habits, and dietary habits to construct a panel dataset for analysis. Finally, a dynamic multivariate panel model (DMPM) [53] is estimated using this dataset.

The greatest advantage of employing DMPM estimation as an analytical method is that it allows for the concise presentation of the underlying assumptions necessary to interpret the relationship between sleep quality and its determinants as causal—even in analyses based on observational studies. These assumptions are represented using a directed acyclic graph (DAG) [23]. A DAG depicts variables as nodes and direct causal relationships between them as directed links. In this study’s analysis, estimation is carried out based on a DAG that identifies causal relationships among key variables primarily through temporal ordering. Accordingly, the validity of the analytical results as causal inferences can be maintained to the extent that the assumption of temporal ordering in causal relationships is accepted.

By leveraging observational causal inference methods and a rich dataset, this paper demonstrates that dietary habits are comparably effective in improving sleep quality, relative to other factors—including both manageable conditions such as exercise routines and bedtime habits, and uncontrollable ones like weather. This finding offers valuable insights for selecting policy measures aimed at promoting public health.

## 2. Materials and Methods

### 2.1. Study Procedure and Participants

The sample obtained through the observational study consisted of approximately 1000 Japanese males and females aged 20 to 80 years, residing in various cities, including Ebetsu City and its surrounding areas in Hokkaido, the Tokyo area, as well as the vicinities of Kyoto City, Nagasaki City, and Miyazaki City. However, since approximately 800 of these individuals were from Ebetsu City and its surrounding areas, this analysis focuses solely on the sample from Ebetsu and its vicinity to ensure data consistency. Using the predominant subsample within the sample helps ensure the integrity of the analysis, but it is important to note that this entails the cost of limiting regional generalizability. Individuals with serious cerebrovascular, cardiac, hepatic, renal, or gastrointestinal diseases, those with legally designated infectious diseases, pregnant females or those who may be pregnant, breastfeeding females, and individuals who had donated blood beyond the specified limits were excluded from the sample based on exclusion criteria. Although the survey covered a wide range of items, the analysis focused on a six-day sleep diary, exercise habits, dietary habits, and health checkup data such as BMI and blood pressure. Data on sleep, exercise, and dietary habits were collected through questionnaires, with dietary habits specifically assessed using the Japanese version of the Food Frequency Questionnaire (FFQ) [54]. Health checkup data were obtained through on-site examinations. The study was conducted from June 2019 to March 2021, and for some survey items, data were collected twice—once in summer and once in winter. A more detailed explanation of the study protocol has been previously reported [55].

This study was approved by the Ethics Committee of the Hokkaido Information University (approval date: 22 April 2019; approval number: 2019-04), and written consent was obtained from the participants. The research was conducted in accordance with the Helsinki Declaration.

### 2.2. Measures

#### 2.2.1. Proxies for Sleep Quality

In this paper, sleep quality is assessed using the following three items: (1) the presence or absence of daytime sleepiness, (2) ease of falling asleep, and (3) feeling refreshed upon waking. Daytime sleepiness was evaluated using a pre-sleep questionnaire that asked, “Since waking up, have you felt extremely sleepy at any point?” with responses coded as 1 = “Yes” or 0 = “No.” Ease of falling asleep was assessed using a post-awakening questionnaire that asked, “How was your ease of falling asleep last night?” with response options of 3 = “I fell asleep easily,” 2 = “It took a little time,” or 1 = “I couldn’t fall asleep.” Feeling refreshed upon waking was also measured using a post-awakening questionnaire, which asked, “Did you feel refreshed upon waking?” with participants selecting one of the following: 3 = “Good,” 2 = “Neutral,” or 1 = “Poor.”

The selection of the scale used in this paper as an outcome variable for assessing sleep quality is primarily constrained by the limitations of available data; nevertheless, this choice is not entirely inappropriate. Objective indicators of sleep quality typically include sleep efficiency, sleep latency, total sleep time, and wake after sleep onset [56]. Although all outcome variables employed in this study are self-reported and therefore subjective, perceived ease of falling asleep corresponds to the subjective equivalent of sleep-onset latency. A sleep latency of no more than 30 min is considered indicative of good sleep quality [57]. Furthermore, the two indicators—ease of falling asleep and ease of waking up—can be interpreted as subjective assessments of deviations from normal sleep patterns, especially with respect to circadian rhythm and homeostasis, which are key factors influencing sleep quality [58]. Daytime sleepiness is not a direct measure of sleep quality but rather a consequence of poor sleep [56]. Unlike objective tools used to measure the severity of excessive daytime sleepiness (EDS), such as the Multiple Sleep Latency Test (MSLT) and the Maintenance of Wakefulness Test (MWT) [59], the outcome variable in this paper captures only the subjective presence or absence of daytime sleepiness, without assessing its intensity.

The validity of the sleep quality measure used in the present analysis was examined in the Supplementary Materials (Figure S1), where its relationship with the Pittsburgh Sleep Quality Index (PSQI) [60,61] was analyzed. The data for the Japanese version of the PSQI [62] were collected via questionnaires administered twice during the observational study—once in summer and once in winter. As shown in Figure S1, the distribution of the within-subject mean values of the three outcome variables used in the analysis shows a statistically significant relationship with PSQI scores, consistent with expectations.

#### 2.2.2. Key Focused Factors Influencing Sleep Quality

This paper estimates a probabilistic model of sleep quality in order to quantitatively evaluate the role that dietary habits play among the various factors affecting sleep quality. Based on existing research, the model incorporates the following six categories as explanatory variables: (1) stress [24,25,26,27,28,29,30], (2) bedtime conditions [31,32], (3) weather conditions [33,34,35,36], (4) physical characteristics [37,38,39,40,41,42,43,44,45], (5) exercise habits [15,46,47,48,49,50,51,52], and (6) dietary habits [10,11,12,13,14,15,16,17,18,19,20,21]. The scales of each factor in the dataset used for the analysis are as follows.

1. Stress: In the sleep diary employed in the present study, six stress-related items were included in the questionnaires administered immediately before bedtime and upon awakening: “I feel relaxed,” “I feel irritated or angry,” “I feel motivated to do things,” “I can concentrate,” “I feel anxious or worried,” and “I feel depressed.” Participants evaluated each item using a four-point scale. Although the Japanese phrasing of these items differs slightly, their content corresponds closely to several stress response items from the Japanese Occupational Stress Questionnaire [63,64].
2. Bedtime conditions: As variables related to bedtime conditions, the analysis utilized items from the post-awakening section of the sleep diary, including bedtime, time in bed, and the presence or absence of caffeine intake, alcohol consumption, and ICT device use before bedtime. Bedtime was converted from the 24 h format recorded in the diary into hourly values relative to midnight (24:00) for analytical purposes.
3. Weather conditions: Weather data were obtained from the historical daily records for Ebetsu City published on the Japan Meteorological Agency’s website [65]. The analysis incorporated daily values for precipitation, average temperature, diurnal temperature range (maximum temperature minus minimum temperature), average wind speed, and sunshine duration on each day of going to bed. Humidity and atmospheric pressure were not included in the published daily data.
4. Physical characteristics: The variables related to physical condition included age, body mass index (BMI), and systolic blood pressure. While BMI and systolic blood pressure were measured during both the summer and winter observation periods, age was recorded at only a single time point. Nonetheless, as the observation period for all study participants was less than one year, any change in age during the study would be limited to a maximum of one year.
5. Exercise habits: Regarding exercise habits, self-reported data on the monthly amount of physical activity for each type of exercise were available at two time points: summer and winter. The types of exercise were categorized into four groups: (i) light walking such as strolling, (ii) brisk walking such as walking for exercise, (iii) light to moderate activities such as golf or gardening, and (iv) vigorous activities such as tennis or jogging. The questionnaire asked participants to report the frequency per month and the duration per session for each type of exercise, from which the monthly amount of activity was calculated. For all types of exercise, the distribution of monthly activity amounts was right-skewed with a median of 0 h/month. Therefore, for each exercise type, a dummy variable was created, taking the value of 1 if the activity amount was greater than zero, and was used in the analysis.
6. Dietary habits: Dietary habit variables were derived from responses to a FFQ administered during the winter phase of the observational study. Since the FFQ inquired about dietary habits over the past year, the responses can also be considered reflective of dietary patterns at the time of the summer sleep diary recordings. Based on the reported intake frequency and portion size for approximately 130 food items, the average daily intake was calculated for each of 12 food group categories (cereals, potatoes, beans, green and yellow vegetables, other vegetables, fruits, mushrooms, seaweeds, seafood, meat, eggs, dairy products). These values were then standardized per 1000 kcal of daily energy intake and used in the analysis.

It should be noted that stress, bedtime conditions, and weather conditions are time-variant variables, while exercise habits and dietary habits are “time-invariant” (i.e., variable that takes a constant value regardless of the observation time). Additionally, physical characteristics are recorded at two time points—summer and winter—and are therefore treated as time-invariant within each seasonal sample. Appendix A provides more detailed descriptions of each variable used in the analysis as well as summary statistics for the sample.

### 2.3. Analyses

**Figure 1:**
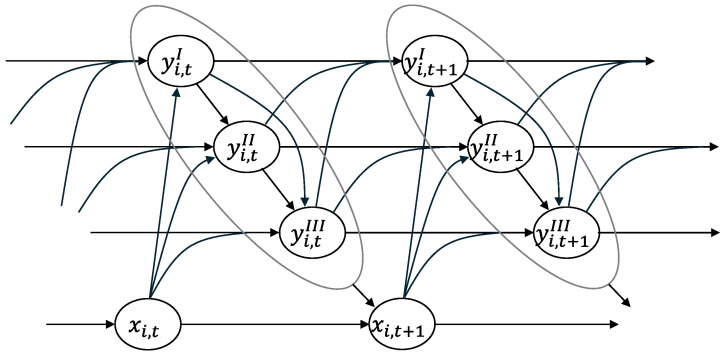
The causal graph depicting the relationships among the three sleep quality indicators ($y^{I}$: daytime sleepiness, $y^{II}$: ease of falling asleep, $y^{III}$: ease of waking up) and contributing factors (x).

This paper estimates a DMPM [53] using sleep diary data to quantitatively assess the effects of various factors thought to influence sleep quality. Figure 1 presents a DAG that summarizes the structure of the DMPM used in the analysis of this paper. The outcome variables of the model—$y_{i,t}^{I}$, $y_{i,t}^{II}$, and $y_{i,t}^{III}$—correspond respectively to the presence or absence of daytime sleepiness, ease of falling asleep, and ease of waking up for individual i on date t. For convenience, the subscript t used to denote dates in the model represents a cycle from one waking time to the next, rather than a cycle from midnight to the following midnight. As indicated by the arrows in the figure, causal relationships among these outcome variables are assumed to follow a temporal order. The serial autocorrelation of outcome variables and the serial cross-correlation between outcome variables in Figure 1 capture the dynamics of sleep quality [23,66,67], and the arrows from outcome variables to factor variables represent feedback effects [67,68,69].

Under a standard lifestyle, where individuals are in bed at night and out of bed from morning to evening, the presence or absence of daytime sleepiness on a given day is affected by the previous day’s daytime sleepiness, the previous day’s ease of falling asleep, and that day’s ease of waking up. The ease of falling asleep on a given day is influenced by the previous day’s ease of falling asleep, that day’s ease of waking up, and that day’s daytime sleepiness. Similarly, the ease of waking up on a given day is affected by the previous day’s ease of waking up, the previous day’s daytime sleepiness, and the previous day’s ease of falling asleep. Even in cases where the day–night cycle is reversed, causal relationships are assumed to follow the same temporal order as shown in Figure 1.

In Figure 1, the determinants of sleep quality faced by individual i on date t are represented as a vector $x_{i,t}$. This vector includes six stress-related factors, five bedtime-related conditions, five meteorological conditions, three physical attributes, four exercise-related habits, and twelve dietary habits. As previously noted, physical attributes, exercise habits, and dietary habits are treated as time-invariant variables. For each outcome variable, the timing of influencing factors is adjusted to reflect the temporal order of causality. For example, within the bedtime conditions, time in bed on a given night affects the next morning’s ease of waking up, but its value from the previous day influences daytime sleepiness and the ease of falling asleep. The specifics of the estimated model are provided in Appendix B.

In order to compare the magnitudes of effects across factor categories, we calculated, for each explanatory variable within the same category, the probability that the outcome would improve when that variable was shifted by one interquartile range in the direction of improvement, and then summed these probabilities for each category. For example, the reduction in the probability of experiencing daytime sleepiness $y^{I}=1$ when all items in factor category A are improved by one interquartile range is given by the following:

(1) $$∆\mathrm{Pr}\left(y^{I}=1|A\right)\equiv \sum_{k\in A}\left|\mathrm{Pr}\left(y^{I}=1|\begin{array}{c} \mathrm{do}\left(x_{k}^{I}={\bar{x}}_{k}\right)\\ x_{-k}^{I} \end{array}\right)-\mathrm{Pr}\left(y^{I}=1|\begin{array}{c} \mathrm{do}\left(x_{k}^{I}={\underline{x}}_{k}\right)\\ x_{-k}^{I} \end{array}\right)\right|$$

where A denotes the set of items in factor category A; $x_{k}^{I}$ is the kth element of the explanatory-variable vector; $x_{-k}^{I}$ is that vector without its kth element; ${\bar{x}}_{k}$ is the first quartile of $x_{k}^{I}$; and ${\underline{x}}_{k}$ is the third quartile of $x_{k}^{I}$. The notation $\mathrm{do}\left(x\right)$, used in the conditioning part of conditional probability, represents the exogenous setting of the variable to a specific value x, and is employed to distinguish it from the probability conditioned merely on the observed value of x [23]. The same approach applies to sleep-onset quality $y^{II}$ and quality of awakening $y^{III}$. Because these outcomes are measured on a three-point scale rather than as a dummy variable like daytime sleepiness $y^{I}$ (Table A1), we use both $∆\mathrm{Pr}\left(y^{I}=1|A\right)$ and $∆\mathrm{Pr}\left(y^{I}=3|A\right)$—the probability changes at the lower and upper ends of the scale for each A—to compare effect sizes across factor categories.

As illustrated by the DAG in Figure 1, the sufficient conditions for identifying Equation (1) are met for all explanatory variables except the time-invariant variables included in vector x. In other words, a set of observed covariates exists that satisfies the back-door criterion [23] for Equation (1). Therefore, in the analysis presented in this paper, the effects of time-variant variables are interpreted as causal—provided that the DAG in Figure 1 accurately represents the underlying causal structure—while the effects of time-invariant variables are interpreted as causal only to the extent that they can reasonably be assumed to be exogenous.

The dataset used in this analysis combines up to six days of sleep diary and meteorological records, to which time-invariant variables from other questionnaires in the same survey were appended for those days. As Equation (1) shows, the effect of each factor category is defined as the change in the outcome variable over the full sample period—excluding the first day, which serves as the initial condition for the vector-autoregressive model—that would result from improving that factor throughout the period. This definition reflects that the factors under comparison include both time-varying and time-invariant variables.

Although the magnitude of improvement for each factor is, in principle, set to its interquartile range, an exception arose for one bedtime-condition item. The dummy variable indicating pre-bedtime caffeine intake has a sample mean below 0.25 (Table A2), yielding an interquartile range of zero. Consequently, for this explanatory variable only, the effect was calculated over an improvement width from 0 (no caffeine) to 1 (caffeine consumed).

The sample was divided into the following four sub-samples, and the DMPM estimation was conducted for each: (a) males in summer, (b) males in winter, (c) females in summer, and (d) females in winter. The model parameters were estimated using Bayesian methods implemented via the R package “dynamite” version 1.5.6 [70].

## 3. Results

**Figure 2:**
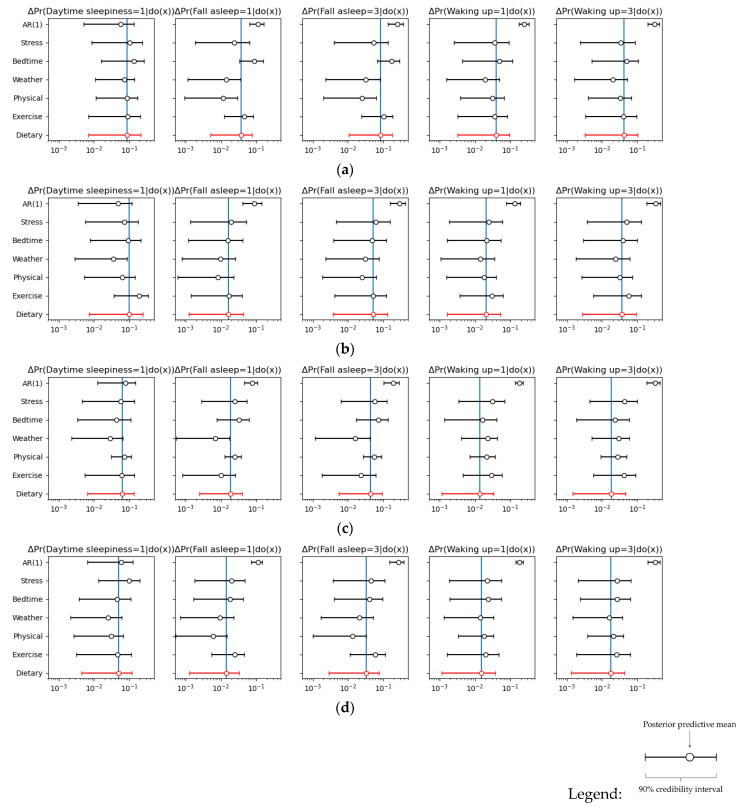
Improvement amounts by outcome and factor category. Hollow circle markers and horizontal lines respectively represent the posterior predictive means and 90% credibility intervals for the metric in Equation (1). Among the various factor categories, the estimated results for dietary habits—which are of particular interest—are highlighted in red. For reference, the blue vertical line indicates the posterior predictive mean improvement attributable to dietary habits. “AR(1)” represents the effect of serial correlation when the value of the outcome variable vector from one period earlier is improved: (**a**) males in summer; (**b**) males in winter; (**c**) females in summer; (**d**) females in winter.

To compare the effects of sleep quality improvement across factor categories, Figure 2 presents the posterior predictive means of the improvement quantities shown in Equation (1), along with their 90% credibility intervals, for each subsample. When all items within a category are improved across the interquartile range of the subsample, the figure shows—by factor category on the vertical axis of each graph—how much the probabilities of experiencing daytime sleepiness (“Daytime sleepiness = 1”), difficulty falling asleep (“Fall asleep = 1”), and poor awakening decrease (“Waking up = 1”), and how much the probabilities of falling asleep easily (“Fall asleep = 3”) and waking up feeling refreshed (“Waking up = 3”) increase. In addition to the six factor categories ranging from stress to dietary habits, the effect of serial autocorrelation in the outcome variable vector is also displayed under the label “AR(1)”.

The magnitude of reduction in the probability of experiencing daytime sleepiness across factor categories varies by gender and season: stress ranges from 6 to 10% points, bedtime conditions from 4 to 13% points, weather conditions from 3 to 7% points, physical characteristics from 3 to 9% points, exercise habits from 5 to 19% points, and dietary habits from 5 to 10% points. In general, males exhibit greater potential for improvement than females across most categories.

With respect to ease of falling asleep, the increases in probability are as follows: stress, 5 to 6% points; bedtime conditions, 4 to 17% points; weather conditions, 2 to 3% points; physical characteristics, 1 to 6% points; exercise habits, 2 to 10% points; and dietary habits, 3 to 8% points. Notably, the effects of bedtime conditions and exercise habits are more pronounced for males during the summer season.

For waking up feeling refreshed, the probability increases are as follows: stress, 3 to 5% points; bedtime conditions, 2 to 5% points; weather conditions, 2 to 3% points; physical characteristics, 2 to 3% points; exercise habits, 3 to 6% points; and dietary habits, 2 to 4% points. Differences due to gender or season are not particularly pronounced in this regard. Across all outcome variables, dietary habits demonstrate effects on sleep quality that are of similar magnitude to those of other factors.

The probability change in the outcome when varying each factor item from the 25th percentile to the 75th percentile—that is, the value inside the absolute value symbols on the right-hand side of Equation (1)—is presented in Supplementary Table S1. When examining the effect of individual factors on daytime sleepiness, under stress-related factors, during winter, both males and females show a higher likelihood of experiencing daytime sleepiness as feelings of irritability increase. Regarding bedtime conditions, during summer for males and during both summer and winter for females, a later bedtime on the previous night is associated with a higher probability of feeling sleepy during the day. Among weather conditions, for men, a greater diurnal temperature range increases the probability of daytime sleepiness in summer but decreases it in winter. Regarding exercise habits, in winter, among men, both very light exercise (e.g., walking) and moderate exercise (e.g., golf or gardening) tend to reduce daytime sleepiness, while light-intensity exercise (e.g., brisk walking) is positively associated with daytime sleepiness. In terms of dietary habits, higher egg intake among males in summer and greater consumption of seafood and dairy products among females in summer show a mitigating effect on daytime sleepiness.

In terms of sleep-onset quality, as expected, bedtime conditions show that in most cases, caffeine intake before bed reduces sleep-onset quality, while alcohol intake improves it. Additionally, a later bedtime or longer time spent in bed the previous day tends to worsen sleep-onset quality. Regarding dietary habits, the intake of green and other vegetables improves sleep-onset quality for females in both summer and winter.

As for waking quality, under stress-related factors, feeling relaxed leads to better wakefulness. For women, feelings of anxiety worsen waking quality. Regarding bedtime conditions, for males in both summer and winter, alcohol intake before bed negatively affects waking quality. Looking at dietary habits, cereal intake among males in summer appears to worsen waking quality.

Among the three outcome variables related to sleep quality, dietary habits have a greater impact on daytime sleepiness than on ease of falling asleep or feeling refreshed upon waking. Compared to daytime sleepiness, ease of falling asleep and feeling refreshed upon waking are more strongly influenced by autocorrelation and cross-correlation over time.

## 4. Discussion

### 4.1. Discussion of the Analysis Results

This study estimated a structural causal model—represented by the DAG in Figure 1—using sleep diary data to clarify the relative importance of dietary habits among the various factors affecting sleep quality. To measure sleep quality, three indicators were employed: presence or absence of daytime sleepiness, ease of falling asleep, and ease of waking up. Factors analyzed for their impact on sleep quality included six stress-related items, five bedtime-related conditions, five weather conditions, three physical characteristics, four exercise habits, and 12 dietary habits.

The analysis revealed that while the magnitude of effect across factor categories varies somewhat depending on gender and season, dietary habits demonstrated impacts that are no smaller than those of other factors. Of course, the six factor categories—from stress to dietary habits—do not equally lend themselves to direct intervention. Weather conditions are given circumstances for individuals, and physical characteristics require long-term efforts to improve. Stress management, too, is not easily tackled. Among the six factors, those that individuals can realistically address through practical lifestyle changes are bedtime conditions, exercise habits, and dietary habits. Even when limited to these three modifiable factors, the effect of dietary habits proved to be no less significant than the other two.

Comparison of the results shown in Table S1 with findings from previous studies reveals the following: In our results, seaweed consumption in males showed a positive effect on wakefulness during both summer and winter, while in females, it had a suppressive effect on daytime sleepiness in the winter season. Additionally, the intake of vegetables—regardless of type, including green and yellow, or other varieties—positively affected ease of falling asleep in females in both summer and winter. These findings are consistent with previous studies indicating a positive association between the consumption of healthy foods and sleep quality [11,15]. However, in males, the intake of green and yellow vegetables in summer and other vegetables in winter showed a negative effect on wakefulness—findings that contradict earlier studies. Although positive correlations between carbohydrate consumption and sleep quality have been reported [12,14], such correlations were not clearly observed in the analysis presented in this paper. The intake of cereals and potatoes showed gender and seasonal differences: in males, cereal consumption had a negative effect on wakefulness in summer, while potato consumption showed a positive effect in winter. Conversely, no effects were observed in females regarding sleep quality. While previous studies have reported a positive relationship between bean intake and sleep quality [19], the findings of the present analysis did not support this association. The negative association between the consumption of processed foods and free-sugar-rich foods with sleep quality [11] could not be verified due to limitations in the dietary data used in this study, i.e., our food classification does not include a category for processed foods.

As noted above, the analytical results presented in this paper include points that do not align with those of existing studies. How should this discrepancy be understood? Existing research [10,11,12,13,14,15,16,17,18,19,20,21] encompasses a wide range of approaches, from those based on clinical data [14,18] to those relying on cross-sectional observational data [19,21]. Studies that use clinical data to examine causal relationships tend to focus on the effects of short-term (several days to several weeks) dietary intake or nutrient consumption, rather than long-term eating habits. On the other hand, research that investigates the effects of long-term dietary patterns typically relies on observational data to estimate correlations. This distinction marks a key difference between existing studies and this paper, and is an important consideration when comparing results.

Furthermore, in explaining the mechanisms underlying the relationship between diet and sleep, substances such as tryptophan, serotonin, and melatonin—which play roles in the circadian rhythm—have drawn attention [20]. However, as dietary components, these substances are distributed across the boundaries of the food categories used in this paper’s analysis. In particular, within categories such as vegetables, fruits, and seafood, there is considerable variation. This fact hinders the ability to draw clear-cut conclusions about the impact of intake by food category on sleep quality.

Finally, gender comparisons suggest that discrepancies with prior research findings are smaller among females than males. This may be partly attributable to gender-based differences in the accuracy of FFQ responses across specific food items [71].

### 4.2. Limitations

This paper has several limitations. The first concerns whether the assumptions necessary to interpret the results as causal relationships are sufficiently acceptable. The validity of treating posterior estimates of effect sizes among factors—as presented in Figure 2 and Table S1—as causal depends on different conditions. For time-variant variables, it relies on the validity of the DAG in Figure 1; for time-invariant variables, it depends on the exogeneity assumption in the regression model. The DAG in Figure 1 simply assumes causal structure based on chronological order. While there seem to be no critical objections to this, one potential criticism may relate not to the ordering itself but to the presence or absence of time lags. The DAG assumes all causal relationships occur within adjacent time periods, yet in reality, some effects may involve longer lags or extend over multiple periods. For instance, a sharp temperature fluctuation yesterday could affect not just today’s sleep but tomorrow’s as well. This illustrates that constructing a valid DAG requires drawing on domain knowledge or expertise. On the other hand, as for the exogeneity assumptions for time-invariant variables, long-term states such as exercise or dietary habits—when treated as external factors affecting daily sleep-related changes recorded in sleep diaries—appear to be largely acceptable.

Secondly, this paper quantitatively evaluated the effects of several factors influencing sleep quality, categorized by type. In doing so, comparisons were made based on the magnitude of impact that changes within the interquartile range of each factor had on sleep quality, as estimated by the model. However, while this approach is appropriate when the factors change independently or autonomously, it is not suitable for precise quantitative comparison when considering real-world situations where strong interrelationships exist among the individual factors—such that changing one inevitably affects others.

Thirdly, aside from the issue of comparing effects based on autonomous changes in individual factors, there is also the problem that interactions between factors are not considered in the analysis. More precisely, while the nonlinearity of the regression model—with a discrete variable as the regressand—does imply definitional interactions among regressors, the interactions themselves are not the subject of estimation. Synergistic effects arising from combinations of factor categories [72,73], such as dietary habits and exercise habits, or exercise habits and stress management, are beyond the scope of this paper’s analysis.

Fourth, this analysis utilizes data from a Food Frequency Questionnaire (FFQ). However, because the data are self-reported and rely on participants’ memory, they are subject to inherent limitations such as recall bias, response bias, social desirability bias, and misclassification ([74] and its references). Moreover, the estimated intake by food category is derived by multiplying the representative values of intake frequency and portion size for each stratum based on FFQ items, which introduces constraints on the precision of the estimation.

In addition to the major points mentioned above, the following limitations can also be noted: the constraints on generalizability due to the regional specificity of the sample; and, due to data limitations, the analysis relies solely on subjective measures of sleep quality as the outcome variable, without incorporating objective indicators such as actual sleep duration or the number of awakenings during time in bed. Sleep quality is a multidimensional construct, and both subjective and objective measures are essential for a comprehensive assessment.

## 5. Conclusions

By estimating a structural causal model using sleep diary data collected during both summer and winter from healthy males and females residing in a specific region of Japan, this study confirms that dietary habits play a non-negligible role among the various factors influencing sleep quality. From the standpoint of public policy, approaches aimed at maintaining and improving sleep quality among healthy individuals have emphasized, in particular, sleep education, cognitive therapy strategies, physical exercise, and dietary improvement [75]. Among these approaches, the present analysis focuses on dietary and exercise habits, and the results reaffirm the significant roles both play in contributing to sleep quality.

## Data Availability

The data obtained from the “Sukoyaka Health Survey” are available in a publicly accessible repository managed by the DNA Data Bank of Japan (DDBJ) Japanese Genotype Phenotype Archive at https://www.ddbj.nig.ac.jp/jga/index-e.html, accessed on 18 February 2025.

## Supplementary Materials

The following supporting information can be downloaded at https://www.mdpi.com/article/10.3390/nu17172787/s1: Figure S1: the relationship between three outcome variables and the Pittsburgh Sleep Quality Index (PSQI); Table S1: the effect of individual factors on sleep quality.

## Abbreviations

The following abbreviations are used in this manuscript:

DAG	Directed acyclic graph
DMPM	Dynamic multivariate panel model
FFQ	Food frequency questionnaire
PSQI	Pittsburgh Sleep Quality Index

## Appendix A

The variables used in the analysis conducted in this paper are listed in Table A1. Their summary statistics are provided in Table A2.

**Table A1:** Variable list.

Variable	Description
(Outcome variables)	
Daytime sleepiness	Indicating daytime sleepiness (1 = Yes, 0 = No)
Fall asleep	1 = Poor sleep onset, 2 = Slight delay in sleep onset, 3 = Good sleep onset
Waking up	1 = Difficult awakening, 2 = Average, 3 = Refreshing awakening
(Stress)	
Relax	The intensity of each of the following states—relaxation, irritability, motivation, concentration, worry, and depressed mood—was assessed both before bedtime and after waking using a four-point scale: 1 = Strongly agree, 2 = Agree, 3 = Disagree, 4 = Strongly disagree
Irritable	
Motivated	
Concentration	
Worried	
Feel down	
(Bedtime conditions)	
Caffeine	Indicating caffeine intake before bedtime (1 = Yes, 0 = No)
Alcohol	Indicating alcohol intake before bedtime (1 = Yes, 0 = No)
ICT	Indicating use of ICT devices before bedtime (1 = Yes, 0 = No)
Bed time	Bedtime (measured in hourly units from 12:00 AM as the reference point)
Time in bed	Time in bed (in hours)
(Weather conditions)	
Rain	Precipitation (mm)
Temperature	Average temperature (°C)
ΔTemperature	Diurnal temperature range (°C)
Wind	Average wind speed (m/s)
Sun light	Sunshine duration (hours)
(Physical characteristics)	
Age	Age
BMI	Body mass index
SBP	Systolic blood pressure
(Exercise habits)	
Exercise 1	Indicating a habit of light physical activity such as walking (1 = Yes, 0 = No)
Exercise 2	Indicating a habit of moderate physical activity such as brisk walking (1 = Yes, 0 = No)
Exercise 3	Indicating a habit of moderate physical activity such as golf or gardening (1 = Yes, 0 = No)
Exercise 4	Indicating a habit of vigorous physical activity such as tennis or jogging (1 = Yes, 0 = No)
(Dietary habits)	
Cereals/Potatoes/Beans/GY vegetables/Other vegetables/Fruits/Mushrooms/Seaweeds/Seafood/Meat/Eggs/Dairy	Intake per 1000 kcal of daily energy consumption (g/kcal)

**Table A2:** Summary statistics.

Variable	Males, Summer (n = 1118)		Males, Winter (n = 1110)		Females, Summer (n = 2893)		Females, Winter (n = 2923)	
	Mean	sd	Mean	sd	Mean	sd	Mean	sd
(Outcome variables)								
Daytime sleepiness	0.42	0.49	0.38	0.49	0.48	0.50	0.43	0.49
Fall asleep	2.64	0.58	2.67	0.54	2.55	0.62	2.55	0.62
Waking up	2.21	0.63	2.20	0.62	2.14	0.65	2.12	0.61
(Stress)								
Relax (before)	3.25	0.82	3.26	0.82	3.15	0.86	3.18	0.83
Relax (after)	3.02	0.86	3.05	0.89	2.85	0.93	2.86	0.92
Irritable (before)	1.36	0.64	1.40	0.66	1.40	0.68	1.43	0.68
Irritable (after)	1.36	0.63	1.42	0.64	1.39	0.66	1.43	0.67
Motivated (before)	2.62	0.90	2.59	0.97	2.36	0.89	2.36	0.89
Motivated (after)	2.85	0.82	2.80	0.89	2.71	0.86	2.71	0.85
Concentration (before)	2.58	0.88	2.56	0.93	2.30	0.85	2.32	0.86
Concentration (after)	2.70	0.84	2.74	0.86	2.53	0.84	2.58	0.85
Worried (before)	1.69	0.87	1.73	0.87	1.90	0.96	1.91	0.95
Worried (after)	1.67	0.85	1.73	0.86	1.86	0.92	1.91	0.94
Feel down (before)	1.40	0.68	1.49	0.72	1.53	0.79	1.54	0.78
Feel down (after)	1.42	0.69	1.47	0.69	1.52	0.77	1.53	0.75
(Bedtime conditions)								
Caffeine	0.15	0.36	0.16	0.37	0.15	0.36	0.17	0.37
Alcohol	0.44	0.50	0.45	0.50	0.25	0.44	0.24	0.43
ICT	0.59	0.49	0.61	0.49	0.64	0.48	0.64	0.48
Bed time	−0.39	1.75	−0.37	1.77	−0.34	1.63	−0.26	1.58
Time in bed	6.80	1.59	6.99	1.54	6.88	1.64	7.10	1.75
(Weather conditions)								
Rain	2.21	5.47	1.83	3.84	2.15	5.52	1.76	3.51
Temperature	18.27	2.93	0.00	5.16	18.34	3.05	0.61	5.17
ΔTemperature	10.07	3.51	8.82	3.57	10.51	3.73	8.71	3.55
Wind	3.29	1.41	3.03	1.47	3.28	1.42	3.05	1.44
Sun light	6.03	4.44	3.08	2.29	6.63	4.55	2.99	2.19
(Physical characteristics)								
Age	53.94	12.43	54.11	12.40	49.82	10.97	50.04	11.06
BMI	23.73	3.59	23.66	3.14	21.46	3.36	21.55	3.37
SBP	128.04	17.87	129.51	17.54	114.47	16.87	112.75	16.52
(Exercise habits)								
Exercise 1	0.40	0.49	0.47	0.50	0.38	0.49	0.42	0.49
Exercise 2	0.31	0.46	0.42	0.49	0.30	0.46	0.31	0.46
Exercise 3	0.47	0.50	0.37	0.48	0.39	0.49	0.31	0.46
Exercise 4	0.31	0.46	0.23	0.42	0.14	0.35	0.16	0.37
(Dietary habits)								
Cereals	183.28	32.38	182.47	32.71	176.14	12.40	175.99	12.64
Potatoes	19.62	4.52	19.58	4.53	23.16	1.83	23.15	1.85
Beans	40.66	23.36	40.36	22.99	43.94	21.16	44.23	21.77
GY vegetables	80.02	28.35	80.37	29.80	88.00	18.36	88.23	18.56
Other vegetables	89.58	19.92	89.42	19.99	98.95	10.71	98.88	10.84
Fruits	63.97	12.60	64.28	12.77	88.02	7.54	88.16	7.78
Mushrooms	7.60	2.62	7.59	2.62	12.15	6.67	12.20	6.69
Seaweeds	5.94	1.88	5.95	1.90	5.06	1.50	5.08	1.54
Seafood	47.71	4.79	47.72	4.71	45.24	3.29	45.16	3.23
Meat	35.23	6.98	35.11	6.79	39.06	11.27	39.14	11.39
Eggs	16.64	1.72	16.70	1.75	19.52	5.76	19.61	5.89
Dairy	81.33	41.43	81.41	42.06	94.79	26.43	94.94	27.42

## Appendix B

This section presents the details of the DMPM estimated in this paper. For convenience, the subscript t used to denote dates is defined as a cycle from one waking time to the next, rather than the conventional midnight-to-midnight cycle. The three outcomes—daytime sleepiness $y_{i,t}^{I}$, ease of sleep onset $y_{i,t}^{II}$, and ease of awakening $y_{i,t}^{III}$—are modeled using Equations (A1)–(A4).

(A1) $$\left\{\begin{array}{c} y_{i,t}^{I}\left|\eta_{i,t}^{I}\right.\;\sim \;\mathrm{Bernoulli}\left({\mathrm{logit}}^{-1}\left(\eta_{i,t}^{I}\right)\right),\\ \eta_{i,t}^{I}=\beta_{AR,1}^{I}y_{i,t-1}^{I}+{\mathrm{Dummy}\left(y_{i,t-1}^{II}\right)}^{\prime }\beta_{AR,2}^{I}+{\mathrm{Dummy}\left(y_{i,t-1}^{III}\right)}^{\prime }\beta_{AR,3}^{I}+x_{I,i,t}^{\prime }\beta^{I}+\nu_{i}^{I}, \end{array}\right.$$

(A2) $$\left\{\begin{array}{c} \mathrm{Pr}\left(y_{i,t}^{II}=j|\eta_{i,t}^{II},\kappa^{II}\right)={\mathrm{logit}}^{-1}\left(\kappa_{j}^{II}-\eta_{i,t}^{II}\right)-{\mathrm{logit}}^{-1}\left(\kappa_{j-1}^{II}-\eta_{i,t}^{II}\right)\;\left(j=1,2,3\right),\\ \eta_{i,t}^{II}=\beta_{AR,1}^{II}y_{i,t}^{I}+{\mathrm{Dummy}\left(y_{i,t-1}^{II}\right)}^{\prime }\beta_{AR,2}^{II}+{\mathrm{Dummy}\left(y_{i,t-1}^{III}\right)}^{\prime }\beta_{AR,3}^{II}+x_{II,i,t}^{\prime }\beta^{II}+\nu_{i}^{II}, \end{array}\right.$$

(A3) $$\left\{\begin{array}{c} \mathrm{Pr}\left(y_{i,t}^{III}=j|\eta_{i,t}^{III},\kappa^{III}\right)={\mathrm{logit}}^{-1}\left(\kappa_{j}^{III}-\eta_{i,t}^{III}\right)-{\mathrm{logit}}^{-1}\left(\kappa_{j-1}^{III}-\eta_{i,t}^{III}\right)\;\left(j=1,2,3\right),\\ \eta_{i,t}^{III}=\beta_{AR,1}^{III}y_{i,t}^{I}+{\mathrm{Dummy}\left(y_{i,t-1}^{II}\right)}^{\prime }\beta_{AR,2}^{III}+{\mathrm{Dummy}\left(y_{i,t-1}^{III}\right)}^{\prime }\beta_{AR,3}^{III}+x_{III,i,t}^{\prime }\beta^{III}+\nu_{i}^{III}, \end{array}\right.$$

(A4) $$\left\{\begin{array}{c} x_{i,t}\left|\eta_{i,t}^{X}\right.\;\sim \;{\mathrm{Link}}^{X}\left(\eta_{i,t}^{X}\right),\\ \eta_{i,t}^{X}=\alpha^{X}+\beta_{AR,1}^{X}y_{i,t-1}^{I}+{\mathrm{Dummy}\left(y_{i,t-1}^{II}\right)}^{\prime }\beta_{AR,2}^{X}+{\mathrm{Dummy}\left(y_{i,t-1}^{III}\right)}^{\prime }\beta_{AR,3}^{X}+\beta^{X}x_{i,t-1}, \end{array}\right.$$

where $\mathrm{Dummy}\left(\;\right)$ refers to the dummy coding function—that is, categorical independent variables are converted into dummy variables for regression analysis. For binary outcome ($y_{i,t}^{I}$), a logistic regression model was employed, while ordinal outcomes ($y_{i,t}^{II}$ and $y_{i,t}^{III}$) were analyzed using ordered logistic regression. Equation (A4) is a nonlinear autoregressive model for time-variant variables, such as stress factors and bedtime conditions. The $\mathrm{Link}\left(\;\right)$ function within the equation represents the distribution defined according to the nature of each variable: for binary variables like caffeine intake, it follows a Bernoulli distribution; for ordinal variables like stress responses, it follows an ordinal logit model; and for continuous variables like bedtime and time in bed, it follows a normal distribution.

While the explanatory-variable vectors $x_{I,i,t}$, $x_{II,i,t}$, and $x_{III,i,t}$ comprises an identical set of variables across Equations (A1)–(A3), the timing of individual variables may vary by equation. This variation reflects the assumption of causal relationships based on temporal sequencing. In Equation (A1) for daytime sleepiness ($y_{i,t}^{I}$), stress levels reported upon awakening on the same day were used as explanatory variables, whereas in Equations (A2) and (A3) for sleep-onset quality ($y_{i,t}^{II}$) and ease of awakening ($y_{i,t}^{III}$), respectively, stress levels reported at bedtime for the corresponding sleep episode were used as explanatory variables. The timing of bedtime conditions used as explanatory variables is offset by one day between the equation for daytime sleepiness and those for sleep-onset and awakening quality.

Equations (A1)–(A3) include individual-specific random effects $\nu_{i}^{I}$, $\nu_{i}^{II}$, and $\nu_{i}^{III}$. The portion of the between-individual variation in the outcome variables that cannot be explained by the selected explanatory variables is captured by these terms.
