# Supplementary material for: How Important Are Dietary Habits Compared to Other Factors for Sleep Quality?—An Analysis Using Data from a Specific Region in Japan
Source: Nutrients. 2025 Aug 27;17(17):2787. doi: 10.3390/nu17172787 (PMC12430039; doi:10.3390/nu17172787)
Supplement: Supplementary file 1 [file nutrients-17-02787-s001.zip › nutrients-3815781-supplementary.pdf]

Supplementary Materials

Figure S1: the relationship between three outcome variables and the Pittsburgh Sleep Quality Index (PSQI)

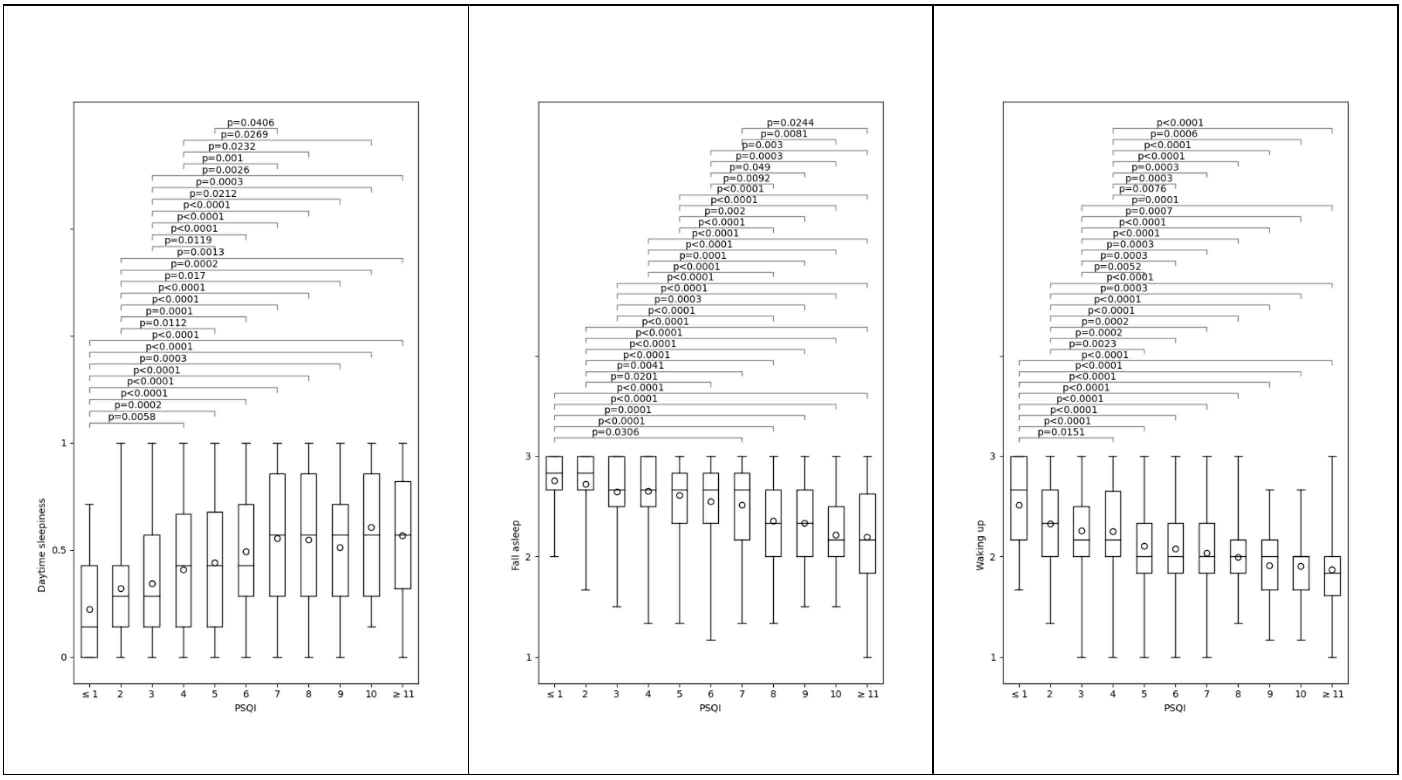

Note: The Dwass, Steel, Critchlow and Fligner all-pairs comparison test on the distribution of within-subject mean values for three sleep-diary outcome variables across PSQI-stratified subsamples. From left to right, the variables are: presence or absence of daytime sleepiness, ease of falling asleep, and quality of awakening. Data from summer and winter were pooled, and p-values are reported whenever distributions differ significantly at the 5% level. Hollow circles represent the mean values of subsamples.

Table S1: the effect of individual factors on sleep quality

Males in summer

	Daytime sleepiness=1			Fall asleep=1			Fall asleep=2			Fall asleep=3			Waking up=1			Waking up=2			Waking up=3		
	Mean	q5	q95	Mean	q5	q95	Mean	q5	q95	Mean	q5	q95	Mean	q5	q95	Mean	q5	q95	Mean	q5	q95
Daytime sleepiness	0.052	-0.005	0.115	0.003	-0.015	0.023	0.003	-0.021	0.027	-0.006	-0.049	0.035	-0.007	-0.031	0.019	-0.001	-0.008	0.004	0.008	-0.018	0.036
Fall asleep	-0.029	-0.090	0.031	-0.082	-0.119	-0.048	-0.096	-0.170	-0.020	0.178	0.099	0.257	-0.096	-0.143	-0.056	0.006	-0.031	0.047	0.090	0.053	0.133
Waking up	-0.059	-0.120	0.000	-0.033	-0.060	-0.011	-0.042	-0.089	-0.007	0.076	0.023	0.136	-0.149	-0.201	-0.103	-0.057	-0.160	0.020	0.206	0.129	0.285
Relax	-0.043	-0.116	0.027	0.012	-0.010	0.036	0.014	-0.012	0.047	-0.026	-0.078	0.021	-0.024	-0.057	0.008	-0.002	-0.017	0.009	0.026	-0.008	0.062
Irritable	0.002	-0.078	0.083	0.030	0.000	0.066	0.033	-0.001	0.083	-0.063	-0.137	0.001	0.038	-0.005	0.085	0.000	-0.019	0.017	-0.037	-0.082	0.005
Motivated	-0.036	-0.119	0.045	-0.031	-0.069	0.004	-0.031	-0.081	0.004	0.062	-0.008	0.141	0.014	-0.030	0.059	0.001	-0.010	0.013	-0.015	-0.062	0.032
Concentration	-0.024	-0.104	0.052	0.000	-0.030	0.032	0.000	-0.041	0.039	0.000	-0.067	0.069	-0.035	-0.084	0.011	0.001	-0.016	0.018	0.034	-0.011	0.082
Worried	0.048	-0.030	0.125	0.011	-0.018	0.041	0.013	-0.022	0.054	-0.024	-0.090	0.040	-0.027	-0.068	0.011	-0.002	-0.018	0.010	0.029	-0.011	0.073
Feel down	-0.032	-0.118	0.056	-0.022	-0.055	0.008	-0.027	-0.076	0.008	0.050	-0.016	0.123	0.031	-0.013	0.078	0.000	-0.016	0.015	-0.031	-0.076	0.013
Caffeine	-0.020	-0.098	0.059	0.070	0.036	0.112	0.065	0.014	0.120	-0.135	-0.208	-0.068	-0.005	-0.040	0.032	-0.002	-0.014	0.005	0.007	-0.031	0.047
Alcohol	-0.009	-0.067	0.049	-0.012	-0.033	0.008	-0.014	-0.045	0.008	0.027	-0.016	0.072	0.035	0.007	0.065	0.001	-0.014	0.016	-0.036	-0.066	-0.008
ICT	-0.042	-0.104	0.021	-0.013	-0.035	0.008	-0.015	-0.046	0.008	0.028	-0.018	0.076	-0.016	-0.044	0.012	-0.001	-0.012	0.006	0.017	-0.012	0.049
Bed time	-0.019	-0.062	0.027	0.014	0.001	0.028	0.016	0.000	0.037	-0.029	-0.060	-0.002	0.019	0.000	0.041	0.001	-0.007	0.011	-0.020	-0.044	0.000
Time in bed	-0.042	-0.079	-0.006	0.027	0.015	0.042	0.031	0.007	0.057	-0.059	-0.089	-0.030	0.011	-0.005	0.029	0.001	-0.005	0.007	-0.012	-0.031	0.006
Rain	0.003	0.001	0.006	0.001	0.000	0.002	0.001	0.000	0.002	-0.002	-0.004	0.000	0.000	-0.002	0.001	0.000	0.000	0.000	0.000	-0.001	0.002
Temperature	0.002	-0.029	0.033	0.010	0.000	0.021	0.011	0.000	0.026	-0.020	-0.044	0.000	0.006	-0.007	0.021	0.000	-0.005	0.003	-0.006	-0.019	0.009
ΔTemperature	0.089	0.036	0.140	0.001	-0.020	0.022	0.002	-0.024	0.029	-0.003	-0.049	0.041	0.013	-0.015	0.042	0.001	-0.006	0.011	-0.014	-0.047	0.015
Wind	0.068	0.024	0.112	-0.010	-0.029	0.007	-0.011	-0.035	0.009	0.020	-0.016	0.061	0.012	-0.009	0.035	0.001	-0.005	0.010	-0.014	-0.039	0.010
Sun light	-0.092	-0.148	-0.034	0.006	-0.016	0.030	0.007	-0.020	0.039	-0.014	-0.064	0.035	-0.021	-0.053	0.010	-0.001	-0.013	0.009	0.022	-0.011	0.057
Age	-0.026	-0.104	0.052	0.016	-0.004	0.038	0.017	-0.005	0.048	-0.033	-0.078	0.010	0.024	-0.002	0.054	0.001	-0.009	0.013	-0.025	-0.056	0.002
BMI	-0.017	-0.064	0.031	0.016	0.003	0.031	0.018	0.002	0.041	-0.035	-0.067	-0.006	0.012	-0.005	0.030	0.001	-0.005	0.007	-0.013	-0.032	0.005
SBP	-0.041	-0.107	0.024	-0.029	-0.051	-0.011	-0.035	-0.072	-0.006	0.064	0.022	0.113	-0.068	-0.099	-0.040	-0.003	-0.034	0.024	0.071	0.040	0.105
Exercise1	-0.013	-0.091	0.063	-0.025	-0.047	-0.006	-0.030	-0.066	-0.004	0.056	0.013	0.104	-0.023	-0.050	0.002	-0.002	-0.014	0.008	0.025	-0.002	0.054
Exercise2	-0.051	-0.136	0.033	0.000	-0.023	0.024	-0.001	-0.030	0.027	0.000	-0.049	0.050	-0.018	-0.047	0.010	-0.002	-0.013	0.007	0.020	-0.010	0.053
Exercise3	0.010	-0.071	0.091	-0.021	-0.044	-0.001	-0.025	-0.060	0.000	0.047	0.003	0.096	0.008	-0.020	0.038	0.000	-0.008	0.007	-0.008	-0.037	0.022
Exercise4	-0.010	-0.095	0.074	0.001	-0.021	0.025	0.001	-0.028	0.028	-0.002	-0.052	0.047	0.001	-0.028	0.031	-0.001	-0.008	0.006	0.000	-0.031	0.031
Cereals	0.039	-0.022	0.099	0.014	0.000	0.030	0.017	0.000	0.041	-0.031	-0.066	0.000	0.031	0.010	0.054	0.002	-0.010	0.016	-0.033	-0.059	-0.010
Potatoes	-0.003	-0.049	0.043	0.000	-0.012	0.011	0.000	-0.016	0.014	0.001	-0.025	0.027	-0.005	-0.020	0.010	0.000	-0.004	0.004	0.005	-0.011	0.021
Beans	0.011	-0.023	0.043	-0.007	-0.017	0.002	-0.008	-0.022	0.002	0.015	-0.004	0.036	0.000	-0.011	0.012	0.000	-0.002	0.003	-0.001	-0.013	0.011
GY vegetables	-0.008	-0.037	0.022	0.002	-0.005	0.010	0.003	-0.006	0.013	-0.005	-0.022	0.011	0.010	0.000	0.021	0.001	-0.003	0.006	-0.011	-0.024	0.000
Other vegetables	-0.002	-0.046	0.042	0.005	-0.006	0.015	0.005	-0.007	0.020	-0.010	-0.034	0.013	-0.002	-0.017	0.013	0.000	-0.004	0.003	0.002	-0.014	0.018
Fruits	0.009	-0.034	0.052	0.001	-0.011	0.012	0.001	-0.013	0.017	-0.002	-0.028	0.024	-0.001	-0.016	0.013	0.000	-0.003	0.004	0.001	-0.015	0.016
Mushrooms	0.008	-0.040	0.056	-0.001	-0.015	0.011	-0.001	-0.017	0.016	0.002	-0.026	0.030	0.003	-0.014	0.021	0.001	-0.003	0.006	-0.004	-0.023	0.014
Seaweeds	0.012	-0.028	0.051	0.004	-0.006	0.015	0.005	-0.007	0.018	-0.009	-0.031	0.013	-0.015	-0.030	-0.001	-0.001	-0.008	0.006	0.016	0.001	0.031
Seafood	0.021	-0.030	0.076	0.010	-0.003	0.025	0.012	-0.003	0.033	-0.021	-0.053	0.006	0.012	-0.007	0.032	0.000	-0.005	0.007	-0.012	-0.032	0.007
Meat	0.001	-0.050	0.053	-0.001	-0.015	0.013	-0.001	-0.018	0.016	0.002	-0.028	0.031	0.006	-0.012	0.024	0.000	-0.004	0.005	-0.006	-0.027	0.013
Eggs	-0.045	-0.084	-0.007	0.011	0.002	0.021	0.014	0.001	0.031	-0.025	-0.048	-0.005	-0.002	-0.015	0.011	0.000	-0.002	0.004	0.001	-0.012	0.015
Dairy	-0.012	-0.051	0.026	-0.003	-0.013	0.007	-0.003	-0.017	0.008	0.006	-0.015	0.028	-0.006	-0.020	0.007	0.000	-0.004	0.003	0.006	-0.007	0.020

Note: For each outcome variable (column headers), the effects of each explanatory factor (row labels) are compared based on the change in the probability of the outcome state (e.g., likelihood of experiencing daytime sleepiness) resulting from a variation within the interquartile range of the explanatory variable values. Cells highlighted in yellow indicate that the credibility interval does not include the value zero. For the notation used in the column and row headers, refer to Table A1 in Appendix A of the paper.

Table S1: the effect of individual factors on sleep quality (continued)

Males in winter

	Daytime sleepiness=1			Fall asleep=1			Fall asleep=2			Fall asleep=3			Waking up=1			Waking up=2			Waking up=3		
	Mean	q5	q95	Mean	q5	q95	Mean	q5	q95	Mean	q5	q95	Mean	q5	q95	Mean	q5	q95	Mean	q5	q95
Daytime sleepiness	-0.043	-0.090	0.005	0.010	-0.003	0.027	0.020	-0.006	0.053	-0.030	-0.073	0.009	0.019	-0.001	0.040	0.013	-0.001	0.033	-0.031	-0.066	0.001
Fall asleep	0.105	0.049	0.160	-0.074	-0.117	-0.035	-0.158	-0.261	-0.036	0.232	0.132	0.326	-0.044	-0.073	-0.020	-0.031	-0.067	-0.004	0.075	0.033	0.122
Waking up	-0.088	-0.150	-0.023	-0.025	-0.048	-0.009	-0.056	-0.109	-0.012	0.081	0.031	0.141	-0.107	-0.151	-0.066	-0.170	-0.290	-0.036	0.277	0.158	0.375
Relax	-0.014	-0.082	0.056	-0.003	-0.021	0.014	-0.007	-0.047	0.030	0.011	-0.043	0.066	-0.045	-0.074	-0.020	-0.038	-0.081	-0.005	0.083	0.034	0.138
Irritable	0.103	0.024	0.184	0.010	-0.010	0.034	0.020	-0.021	0.071	-0.031	-0.096	0.031	0.009	-0.022	0.040	0.006	-0.018	0.033	-0.014	-0.069	0.039
Motivated	-0.004	-0.092	0.082	-0.004	-0.032	0.022	-0.008	-0.067	0.046	0.013	-0.065	0.093	0.066	0.030	0.107	0.047	0.008	0.097	-0.113	-0.182	-0.050
Concentration	-0.034	-0.120	0.048	0.016	-0.010	0.047	0.031	-0.021	0.095	-0.048	-0.130	0.032	-0.054	-0.097	-0.015	-0.037	-0.091	-0.002	0.091	0.025	0.167
Worried	-0.072	-0.143	0.003	-0.010	-0.034	0.012	-0.020	-0.071	0.024	0.029	-0.035	0.098	0.000	-0.029	0.031	0.000	-0.030	0.031	-0.001	-0.059	0.056
Feel down	0.029	-0.055	0.110	0.007	-0.017	0.032	0.014	-0.036	0.067	-0.020	-0.092	0.049	0.007	-0.026	0.042	0.005	-0.020	0.036	-0.013	-0.074	0.044
Caffeine	-0.052	-0.131	0.031	0.005	-0.013	0.026	0.008	-0.030	0.051	-0.013	-0.072	0.042	-0.031	-0.058	-0.006	-0.028	-0.070	-0.001	0.059	0.009	0.116
Alcohol	-0.026	-0.082	0.030	-0.023	-0.042	-0.008	-0.047	-0.094	-0.009	0.070	0.026	0.122	0.024	0.003	0.047	0.017	0.000	0.041	-0.041	-0.079	-0.005
ICT	-0.054	-0.117	0.008	-0.004	-0.019	0.010	-0.009	-0.042	0.020	0.013	-0.029	0.058	-0.014	-0.035	0.007	-0.010	-0.033	0.004	0.024	-0.011	0.063
Bed time	0.071	0.027	0.117	-0.002	-0.011	0.007	-0.003	-0.022	0.014	0.005	-0.021	0.031	0.012	-0.001	0.026	0.009	-0.001	0.026	-0.021	-0.048	0.002
Time in bed	-0.013	-0.052	0.026	0.017	0.007	0.029	0.033	0.008	0.061	-0.050	-0.080	-0.023	0.007	-0.007	0.022	0.005	-0.005	0.020	-0.013	-0.039	0.011
Rain	0.002	-0.011	0.014	0.001	-0.003	0.004	0.002	-0.006	0.010	-0.002	-0.013	0.009	-0.002	-0.007	0.004	-0.001	-0.006	0.003	0.003	-0.006	0.012
Temperature	0.019	-0.020	0.059	-0.003	-0.013	0.005	-0.006	-0.026	0.011	0.009	-0.015	0.036	0.001	-0.012	0.013	0.002	-0.007	0.013	-0.003	-0.024	0.018
ΔTemperature	-0.039	-0.073	-0.006	0.009	0.000	0.020	0.018	-0.001	0.043	-0.026	-0.057	0.001	0.002	-0.012	0.016	0.001	-0.011	0.013	-0.003	-0.028	0.022
Wind	0.017	-0.025	0.059	0.001	-0.010	0.012	0.002	-0.022	0.027	-0.003	-0.037	0.031	-0.001	-0.018	0.015	-0.001	-0.015	0.012	0.003	-0.025	0.032
Sun light	0.006	-0.033	0.045	-0.001	-0.014	0.010	-0.002	-0.028	0.022	0.004	-0.030	0.040	-0.006	-0.024	0.010	-0.004	-0.019	0.008	0.010	-0.018	0.040
Age	0.010	-0.074	0.093	-0.005	-0.019	0.008	-0.010	-0.040	0.018	0.014	-0.026	0.056	0.004	-0.016	0.024	0.003	-0.012	0.020	-0.007	-0.042	0.027
BMI	-0.023	-0.089	0.043	0.002	-0.009	0.013	0.003	-0.019	0.026	-0.005	-0.036	0.026	-0.002	-0.016	0.013	-0.001	-0.014	0.010	0.003	-0.022	0.028
SBP	-0.034	-0.101	0.034	0.007	-0.004	0.020	0.013	-0.008	0.040	-0.020	-0.055	0.011	-0.019	-0.035	-0.003	-0.014	-0.035	-0.001	0.033	0.005	0.065
Exercise1	-0.095	-0.183	-0.007	-0.011	-0.026	0.003	-0.022	-0.058	0.005	0.033	-0.008	0.076	-0.006	-0.026	0.015	-0.005	-0.026	0.010	0.011	-0.024	0.049
Exercise2	0.103	0.007	0.200	-0.008	-0.025	0.007	-0.017	-0.056	0.013	0.026	-0.020	0.075	-0.031	-0.054	-0.010	-0.025	-0.059	-0.003	0.056	0.017	0.101
Exercise3	-0.127	-0.216	-0.038	-0.001	-0.016	0.014	-0.003	-0.037	0.027	0.005	-0.040	0.051	0.021	-0.001	0.044	0.014	-0.001	0.036	-0.034	-0.073	0.001
Exercise4	-0.072	-0.176	0.032	0.007	-0.009	0.026	0.013	-0.020	0.052	-0.021	-0.074	0.030	-0.011	-0.033	0.012	-0.009	-0.034	0.008	0.020	-0.021	0.064
Cereals	0.025	-0.044	0.094	-0.007	-0.019	0.003	-0.014	-0.041	0.007	0.022	-0.010	0.055	0.003	-0.013	0.018	0.002	-0.010	0.015	-0.004	-0.032	0.023
Potatoes	-0.034	-0.086	0.018	-0.001	-0.010	0.008	-0.001	-0.020	0.017	0.002	-0.024	0.028	-0.015	-0.028	-0.002	-0.010	-0.026	0.000	0.025	0.003	0.049
Beans	-0.015	-0.056	0.025	0.003	-0.002	0.010	0.007	-0.005	0.022	-0.011	-0.030	0.007	0.008	0.000	0.017	0.006	0.000	0.016	-0.014	-0.031	0.001
GY vegetables	-0.015	-0.049	0.019	-0.002	-0.008	0.003	-0.004	-0.016	0.005	0.006	-0.008	0.022	-0.001	-0.008	0.006	0.000	-0.006	0.005	0.001	-0.011	0.013
Other vegetables	-0.010	-0.062	0.041	-0.001	-0.010	0.007	-0.003	-0.021	0.015	0.004	-0.021	0.030	0.016	0.004	0.029	0.011	0.001	0.026	-0.027	-0.049	-0.007
Fruits	0.020	-0.028	0.067	0.002	-0.005	0.009	0.004	-0.010	0.021	-0.006	-0.028	0.015	-0.001	-0.013	0.009	0.000	-0.008	0.008	0.002	-0.017	0.020
Mushrooms	0.022	-0.034	0.077	-0.001	-0.011	0.008	-0.001	-0.020	0.017	0.002	-0.023	0.029	-0.007	-0.022	0.005	-0.004	-0.016	0.004	0.012	-0.010	0.034
Seaweeds	-0.011	-0.058	0.034	0.002	-0.004	0.009	0.004	-0.009	0.020	-0.006	-0.028	0.013	-0.010	-0.021	0.000	-0.007	-0.019	0.000	0.018	0.000	0.036
Seafood	-0.011	-0.072	0.052	0.004	-0.006	0.015	0.007	-0.012	0.030	-0.011	-0.042	0.018	0.010	-0.004	0.025	0.007	-0.003	0.021	-0.017	-0.042	0.007
Meat	-0.002	-0.064	0.060	0.018	0.008	0.032	0.038	0.009	0.070	-0.056	-0.090	-0.025	-0.005	-0.019	0.008	-0.003	-0.016	0.006	0.008	-0.014	0.032
Eggs	-0.034	-0.072	0.004	-0.005	-0.014	0.001	-0.010	-0.029	0.003	0.016	-0.005	0.040	0.000	-0.010	0.009	0.000	-0.007	0.008	0.000	-0.016	0.016
Dairy	0.008	-0.034	0.048	-0.002	-0.009	0.004	-0.003	-0.018	0.010	0.005	-0.014	0.025	0.002	-0.006	0.011	0.002	-0.005	0.010	-0.004	-0.020	0.012

Note: For each outcome variable (column headers), the effects of each explanatory factor (row labels) are compared based on the change in the probability of the outcome state (e.g., likelihood of experiencing daytime sleepiness) resulting from a variation within the interquartile range of the explanatory variable values. Cells highlighted in yellow indicate that the credibility interval does not include the value zero. For the notation used in the column and row headers, refer to Table A1 in Appendix A of the paper.

Table S1: the effect of individual factors on sleep quality (continued)

Females in summer

	Daytime sleepiness=1			Fall asleep=1			Fall asleep=2			Fall asleep=3			Waking up=1			Waking up=2			Waking up=3		
	Mean	q5	q95	Mean	q5	q95	Mean	q5	q95	Mean	q5	q95	Mean	q5	q95	Mean	q5	q95	Mean	q5	q95
Daytime sleepiness	0.102	0.056	0.151	0.011	0.000	0.023	0.014	0.000	0.035	-0.025	-0.053	-0.001	-0.002	-0.016	0.012	-0.001	-0.008	0.004	0.003	-0.016	0.023
Fall asleep	0.006	-0.029	0.041	-0.082	-0.110	-0.052	-0.118	-0.202	-0.022	0.200	0.110	0.275	-0.077	-0.102	-0.053	-0.023	-0.055	0.000	0.100	0.060	0.136
Waking up	-0.031	-0.072	0.009	-0.008	-0.021	0.005	-0.010	-0.032	0.006	0.018	-0.011	0.049	-0.113	-0.141	-0.085	-0.094	-0.181	-0.015	0.207	0.117	0.281
Relax	0.026	-0.028	0.079	0.001	-0.011	0.012	0.001	-0.017	0.019	-0.001	-0.029	0.026	-0.028	-0.047	-0.011	-0.011	-0.028	-0.001	0.040	0.014	0.069
Irritable	0.001	-0.044	0.047	0.003	-0.011	0.017	0.004	-0.015	0.025	-0.007	-0.040	0.026	-0.016	-0.037	0.004	-0.006	-0.019	0.001	0.022	-0.005	0.053
Motivated	-0.001	-0.051	0.048	0.013	-0.003	0.030	0.017	-0.004	0.047	-0.029	-0.071	0.007	-0.014	-0.039	0.009	-0.004	-0.017	0.003	0.019	-0.012	0.052
Concentration	-0.056	-0.103	-0.008	0.003	-0.014	0.020	0.004	-0.018	0.028	-0.007	-0.046	0.030	-0.014	-0.038	0.009	-0.005	-0.019	0.003	0.019	-0.012	0.054
Worried	0.046	-0.006	0.099	-0.007	-0.024	0.009	-0.009	-0.036	0.012	0.016	-0.021	0.054	0.048	0.021	0.078	0.013	0.000	0.034	-0.061	-0.100	-0.025
Feel down	0.019	-0.031	0.067	0.012	-0.004	0.029	0.015	-0.005	0.042	-0.027	-0.065	0.010	0.000	-0.023	0.022	0.000	-0.010	0.008	0.001	-0.029	0.031
Caffeine	0.008	-0.039	0.056	0.023	0.007	0.040	0.027	0.004	0.059	-0.050	-0.091	-0.017	-0.005	-0.025	0.016	-0.002	-0.013	0.005	0.007	-0.020	0.036
Alcohol	-0.004	-0.046	0.038	-0.013	-0.026	-0.001	-0.018	-0.043	-0.001	0.031	0.004	0.063	-0.009	-0.026	0.008	-0.004	-0.015	0.002	0.013	-0.010	0.038
ICT	0.054	0.016	0.091	-0.005	-0.016	0.006	-0.007	-0.025	0.008	0.012	-0.014	0.039	0.000	-0.016	0.016	0.000	-0.007	0.006	0.000	-0.022	0.022
Bed time	0.017	-0.006	0.040	0.010	0.004	0.017	0.013	0.002	0.028	-0.023	-0.042	-0.008	0.016	0.006	0.027	0.005	0.000	0.014	-0.021	-0.037	-0.007
Time in bed	-0.054	-0.076	-0.033	0.018	0.010	0.026	0.023	0.005	0.042	-0.041	-0.061	-0.022	-0.005	-0.014	0.004	-0.002	-0.006	0.001	0.006	-0.005	0.019
Rain	0.001	-0.001	0.003	0.000	0.000	0.001	0.000	0.000	0.001	-0.001	-0.002	0.001	-0.001	-0.001	0.000	0.000	-0.001	0.000	0.001	0.000	0.002
Temperature	0.000	-0.020	0.020	0.003	-0.002	0.009	0.003	-0.004	0.012	-0.006	-0.020	0.006	-0.004	-0.011	0.004	-0.002	-0.007	0.001	0.006	-0.004	0.017
ΔTemperature	-0.025	-0.060	0.010	0.001	-0.010	0.012	0.002	-0.013	0.019	-0.003	-0.028	0.022	-0.017	-0.035	-0.002	-0.005	-0.015	0.000	0.022	0.002	0.045
Wind	-0.020	-0.051	0.012	-0.001	-0.011	0.008	-0.001	-0.016	0.012	0.003	-0.020	0.026	-0.008	-0.023	0.005	-0.003	-0.009	0.002	0.011	-0.007	0.030
Sun light	0.024	-0.018	0.066	-0.005	-0.019	0.009	-0.007	-0.028	0.011	0.011	-0.020	0.044	0.008	-0.012	0.029	0.003	-0.004	0.012	-0.010	-0.038	0.015
Age	0.041	0.008	0.072	0.017	0.008	0.027	0.022	0.004	0.043	-0.039	-0.063	-0.017	-0.010	-0.021	0.001	-0.003	-0.010	0.000	0.013	-0.002	0.029
BMI	0.008	-0.017	0.033	0.005	0.000	0.012	0.007	0.000	0.019	-0.012	-0.028	0.001	-0.019	-0.029	-0.009	-0.006	-0.015	0.000	0.025	0.011	0.040
SBP	0.024	-0.008	0.056	0.002	-0.006	0.010	0.003	-0.008	0.015	-0.005	-0.023	0.013	0.007	-0.003	0.018	0.002	-0.001	0.009	-0.009	-0.025	0.005
Exercise1	-0.044	-0.088	0.000	0.014	0.003	0.026	0.017	0.002	0.040	-0.031	-0.060	-0.006	-0.003	-0.018	0.012	-0.001	-0.008	0.004	0.005	-0.015	0.025
Exercise2	0.007	-0.041	0.054	-0.004	-0.016	0.007	-0.006	-0.026	0.009	0.011	-0.016	0.039	-0.003	-0.018	0.013	-0.001	-0.008	0.005	0.004	-0.018	0.025
Exercise3	-0.016	-0.059	0.027	-0.003	-0.014	0.007	-0.004	-0.020	0.010	0.007	-0.017	0.032	-0.011	-0.026	0.004	-0.004	-0.013	0.001	0.015	-0.005	0.037
Exercise4	0.002	-0.059	0.062	-0.006	-0.021	0.009	-0.009	-0.033	0.010	0.015	-0.018	0.050	-0.012	-0.032	0.009	-0.005	-0.018	0.002	0.017	-0.011	0.046
Cereals	-0.004	-0.032	0.025	-0.006	-0.014	0.001	-0.008	-0.022	0.001	0.014	-0.002	0.033	0.001	-0.009	0.012	0.000	-0.004	0.005	-0.001	-0.015	0.012
Potatoes	-0.013	-0.039	0.012	0.002	-0.004	0.008	0.002	-0.006	0.011	-0.004	-0.018	0.009	0.003	-0.005	0.011	0.001	-0.002	0.005	-0.004	-0.015	0.007
Beans	-0.004	-0.021	0.013	0.002	-0.002	0.006	0.002	-0.003	0.010	-0.004	-0.014	0.005	-0.005	-0.011	0.001	-0.002	-0.005	0.000	0.006	-0.002	0.015
GY vegetables	-0.002	-0.022	0.018	-0.009	-0.016	-0.004	-0.012	-0.024	-0.002	0.021	0.008	0.036	-0.005	-0.013	0.002	-0.001	-0.005	0.001	0.006	-0.003	0.017
Other vegetables	-0.012	-0.032	0.009	-0.005	-0.011	0.000	-0.007	-0.018	0.000	0.012	0.000	0.026	-0.006	-0.014	0.001	-0.002	-0.007	0.000	0.008	-0.002	0.019
Fruits	0.005	-0.017	0.028	0.004	-0.001	0.009	0.005	-0.002	0.014	-0.008	-0.021	0.003	0.009	0.001	0.017	0.003	0.000	0.007	-0.011	-0.023	-0.001
Mushrooms	0.004	-0.015	0.023	0.002	-0.002	0.007	0.003	-0.002	0.011	-0.005	-0.016	0.004	0.000	-0.007	0.007	0.000	-0.003	0.002	0.000	-0.009	0.010
Seaweeds	-0.006	-0.020	0.006	0.000	-0.003	0.003	0.000	-0.004	0.004	0.000	-0.007	0.006	0.003	-0.001	0.007	0.001	0.000	0.003	-0.004	-0.010	0.002
Seafood	-0.029	-0.053	-0.004	-0.003	-0.009	0.003	-0.004	-0.014	0.004	0.007	-0.007	0.021	0.001	-0.007	0.010	0.000	-0.003	0.004	-0.001	-0.013	0.010
Meat	0.020	-0.002	0.043	0.003	-0.003	0.008	0.003	-0.004	0.013	-0.006	-0.019	0.006	-0.006	-0.014	0.002	-0.002	-0.007	0.000	0.008	-0.002	0.019
Eggs	0.003	-0.009	0.015	-0.004	-0.008	-0.001	-0.006	-0.013	-0.001	0.010	0.002	0.019	0.001	-0.003	0.006	0.000	-0.001	0.002	-0.002	-0.007	0.004
Dairy	-0.020	-0.035	-0.006	-0.003	-0.006	0.001	-0.004	-0.010	0.000	0.006	-0.001	0.015	-0.001	-0.006	0.004	0.000	-0.003	0.001	0.001	-0.005	0.008

Note: For each outcome variable (column headers), the effects of each explanatory factor (row labels) are compared based on the change in the probability of the outcome state (e.g., likelihood of experiencing daytime sleepiness) resulting from a variation within the interquartile range of the explanatory variable values. Cells highlighted in yellow indicate that the credibility interval does not include the value zero. For the notation used in the column and row headers, refer to Table A1 in Appendix A of the paper.

Table S1: the effect of individual factors on sleep quality (continued)

Females in winter

	Daytime sleepiness=1			Fall asleep=1			Fall asleep=2			Fall asleep=3			Waking up=1			Waking up=2			Waking up=3		
	Mean	q5	q95	Mean	q5	q95	Mean	q5	q95	Mean	q5	q95	Mean	q5	q95	Mean	q5	q95	Mean	q5	q95
Daytime sleepiness	0.064	0.024	0.109	0.003	-0.007	0.014	0.004	-0.010	0.019	-0.007	-0.031	0.016	-0.003	-0.018	0.013	-0.001	-0.005	0.002	0.003	-0.015	0.022
Fall asleep	0.006	-0.029	0.040	-0.107	-0.140	-0.072	-0.155	-0.258	-0.031	0.262	0.147	0.351	-0.062	-0.084	-0.042	-0.009	-0.028	0.005	0.072	0.045	0.098
Waking up	-0.013	-0.054	0.028	-0.008	-0.021	0.005	-0.010	-0.030	0.006	0.017	-0.010	0.048	-0.131	-0.162	-0.100	-0.106	-0.200	-0.019	0.237	0.144	0.313
Relax	0.054	0.000	0.107	-0.007	-0.020	0.005	-0.009	-0.029	0.006	0.016	-0.012	0.045	-0.034	-0.055	-0.015	-0.008	-0.022	0.001	0.042	0.018	0.069
Irritable	0.065	0.020	0.110	0.001	-0.013	0.016	0.002	-0.018	0.022	-0.003	-0.036	0.029	0.053	0.028	0.080	0.006	-0.006	0.021	-0.059	-0.088	-0.032
Motivated	-0.025	-0.071	0.023	-0.013	-0.030	0.003	-0.016	-0.044	0.004	0.028	-0.007	0.070	-0.010	-0.034	0.015	-0.002	-0.010	0.003	0.012	-0.017	0.041
Concentration	-0.031	-0.079	0.017	0.016	-0.001	0.034	0.020	-0.001	0.051	-0.036	-0.079	0.001	-0.011	-0.036	0.014	-0.002	-0.010	0.002	0.013	-0.016	0.043
Worried	0.017	-0.038	0.072	0.012	-0.004	0.029	0.015	-0.004	0.042	-0.027	-0.066	0.009	0.031	0.007	0.056	0.004	-0.002	0.016	-0.035	-0.066	-0.008
Feel down	0.014	-0.037	0.065	0.007	-0.009	0.024	0.009	-0.012	0.035	-0.016	-0.053	0.021	-0.018	-0.043	0.006	-0.003	-0.012	0.002	0.022	-0.007	0.052
Caffeine	0.034	-0.010	0.080	0.022	0.007	0.039	0.025	0.004	0.054	-0.047	-0.086	-0.015	-0.011	-0.032	0.009	-0.003	-0.011	0.001	0.014	-0.010	0.040
Alcohol	0.016	-0.029	0.061	-0.025	-0.039	-0.013	-0.034	-0.066	-0.007	0.059	0.026	0.096	0.018	-0.001	0.040	0.002	-0.002	0.009	-0.020	-0.044	0.002
ICT	0.015	-0.025	0.053	-0.007	-0.018	0.005	-0.009	-0.028	0.005	0.015	-0.010	0.044	0.005	-0.011	0.023	0.001	-0.003	0.004	-0.006	-0.026	0.014
Bed time	0.011	-0.017	0.039	0.008	0.001	0.015	0.010	0.001	0.023	-0.017	-0.035	-0.002	0.020	0.009	0.033	0.004	-0.001	0.011	-0.024	-0.040	-0.010
Time in bed	-0.051	-0.074	-0.028	0.021	0.014	0.029	0.027	0.006	0.046	-0.048	-0.068	-0.027	-0.013	-0.024	-0.004	-0.002	-0.007	0.001	0.016	0.004	0.028
Rain	0.010	0.000	0.021	-0.005	-0.009	-0.001	-0.006	-0.013	-0.001	0.010	0.002	0.020	-0.006	-0.011	-0.001	-0.001	-0.003	0.000	0.007	0.001	0.013
Temperature	-0.030	-0.055	-0.005	0.006	0.000	0.012	0.008	0.000	0.019	-0.013	-0.030	0.001	-0.003	-0.014	0.007	0.000	-0.003	0.002	0.004	-0.008	0.016
ΔTemperature	0.004	-0.019	0.027	0.004	-0.004	0.012	0.005	-0.005	0.017	-0.009	-0.028	0.009	0.000	-0.012	0.012	0.000	-0.003	0.002	0.001	-0.013	0.014
Wind	0.021	-0.005	0.048	-0.003	-0.012	0.005	-0.004	-0.017	0.007	0.007	-0.012	0.027	0.003	-0.009	0.016	0.000	-0.002	0.004	-0.004	-0.019	0.011
Sun light	0.012	-0.012	0.037	-0.010	-0.019	-0.001	-0.012	-0.028	-0.001	0.022	0.002	0.044	-0.006	-0.019	0.007	-0.001	-0.004	0.002	0.006	-0.008	0.022
Age	0.000	-0.034	0.033	0.009	0.001	0.017	0.011	0.001	0.026	-0.020	-0.041	-0.002	-0.010	-0.021	0.002	-0.002	-0.006	0.001	0.011	-0.002	0.025
BMI	0.023	-0.003	0.050	0.001	-0.005	0.007	0.001	-0.007	0.010	-0.002	-0.016	0.011	-0.003	-0.013	0.006	-0.001	-0.003	0.001	0.004	-0.007	0.015
SBP	0.003	-0.030	0.037	-0.006	-0.014	0.002	-0.007	-0.021	0.002	0.013	-0.005	0.033	-0.005	-0.017	0.007	-0.001	-0.004	0.001	0.006	-0.008	0.020
Exercise1	-0.015	-0.061	0.030	0.005	-0.006	0.017	0.006	-0.007	0.024	-0.012	-0.037	0.012	-0.002	-0.018	0.014	-0.001	-0.005	0.002	0.003	-0.016	0.022
Exercise2	-0.010	-0.064	0.045	0.003	-0.008	0.015	0.004	-0.011	0.022	-0.007	-0.034	0.018	-0.010	-0.028	0.008	-0.002	-0.008	0.001	0.012	-0.009	0.033
Exercise3	0.005	-0.046	0.054	-0.010	-0.021	0.001	-0.013	-0.034	0.001	0.022	-0.003	0.051	0.019	0.001	0.038	0.002	-0.002	0.009	-0.021	-0.042	-0.001
Exercise4	0.005	-0.055	0.065	-0.023	-0.038	-0.010	-0.032	-0.067	-0.006	0.056	0.020	0.096	-0.023	-0.042	-0.003	-0.006	-0.018	0.001	0.028	0.003	0.056
Cereals	-0.013	-0.045	0.019	-0.002	-0.009	0.005	-0.002	-0.013	0.007	0.004	-0.012	0.021	0.002	-0.009	0.012	0.000	-0.002	0.003	-0.002	-0.015	0.010
Potatoes	-0.007	-0.033	0.019	0.000	-0.006	0.006	0.000	-0.008	0.008	0.000	-0.013	0.013	0.003	-0.006	0.012	0.000	-0.001	0.002	-0.003	-0.013	0.007
Beans	-0.012	-0.029	0.006	-0.004	-0.008	0.000	-0.005	-0.012	0.000	0.008	-0.001	0.019	-0.003	-0.010	0.003	-0.001	-0.002	0.001	0.004	-0.003	0.011
GY vegetables	0.006	-0.015	0.027	-0.007	-0.012	-0.002	-0.008	-0.018	-0.001	0.015	0.003	0.028	-0.001	-0.009	0.006	0.000	-0.002	0.001	0.001	-0.007	0.010
Other vegetables	-0.005	-0.027	0.016	-0.006	-0.011	0.000	-0.007	-0.017	0.000	0.012	0.000	0.026	-0.007	-0.015	0.000	-0.001	-0.004	0.001	0.009	0.000	0.018
Fruits	0.010	-0.014	0.033	0.000	-0.005	0.005	0.000	-0.007	0.007	-0.001	-0.013	0.011	0.001	-0.006	0.010	0.000	-0.001	0.002	-0.002	-0.011	0.007
Mushrooms	-0.001	-0.021	0.018	0.001	-0.003	0.006	0.002	-0.004	0.008	-0.003	-0.013	0.007	-0.001	-0.008	0.006	0.000	-0.002	0.001	0.001	-0.007	0.009
Seaweeds	-0.018	-0.033	-0.003	0.002	-0.001	0.004	0.002	-0.001	0.007	-0.004	-0.010	0.002	0.001	-0.003	0.005	0.000	0.000	0.001	-0.001	-0.006	0.003
Seafood	-0.012	-0.040	0.016	-0.001	-0.007	0.005	-0.001	-0.010	0.007	0.002	-0.012	0.016	-0.004	-0.014	0.005	-0.001	-0.003	0.001	0.005	-0.006	0.016
Meat	0.010	-0.014	0.035	0.002	-0.003	0.007	0.003	-0.004	0.011	-0.005	-0.017	0.007	0.004	-0.004	0.012	0.001	-0.001	0.003	-0.005	-0.014	0.004
Eggs	0.001	-0.012	0.013	0.001	-0.002	0.004	0.001	-0.002	0.006	-0.002	-0.009	0.004	0.001	-0.003	0.006	0.000	-0.001	0.001	-0.001	-0.006	0.003
Dairy	0.004	-0.011	0.019	0.000	-0.003	0.003	0.000	-0.005	0.004	0.001	-0.006	0.008	-0.004	-0.009	0.001	-0.001	-0.002	0.000	0.004	-0.001	0.011

Note: For each outcome variable (column headers), the effects of each explanatory factor (row labels) are compared based on the change in the probability of the outcome state (e.g., likelihood of experiencing daytime sleepiness) resulting from a variation within the interquartile range of the explanatory variable values. Cells highlighted in yellow indicate that the credibility interval does not include the value zero. For the notation used in the column and row headers, refer to Table A1 in Appendix A of the paper.
